# Fire severity and soil erosion susceptibility mapping using multi-temporal Earth Observation data: The case of Mati fatal wildfire in Eastern Attica, Greece

**DOI:** 10.1016/j.catena.2019.104320

**Published:** 2020-04

**Authors:** Nikolaos Efthimiou, Emmanouil Psomiadis, Panos Panagos

**Affiliations:** a European Commission, Joint Research Centre (JRC), Ispra, Italy; b Faculty of Environmental Sciences, Czech University of Life Sciences Prague, Kamýcká 129, Praha – Suchdol 165 00, Czech Republic; c Agricultural University of Athens, Department of Natural Resources Management and Agricultural Engineering, Laboratory of Mineralogy and Geology, 75 Iera Odos str., 118 55 Athens, Greece

**Keywords:** Mediterranean, RUSLE, Mati Attika, Remote sensing, Wildlife-urban interface

## Abstract

In recent years, forest fires have increased in terms of frequency, extent and intensity, especially in Mediterranean countries. Climate characteristics and anthropogenic disturbances lead forest environments to display high vulnerability to wildfires, with their sustainability being threatened by the loss of vegetation, changes on soil properties, and increased soil loss rates. Moreover, wildfires are a great threat to property and human life, especially in Wildland-Urban Interface (WUI) areas. In light of the impacts and trends mentioned above, this study aims to assess the impact of the Mati, Attika wildfire on soil erosion. The event caused 102 fatalities, inducing severe consequences to the local infrastructure network; economy; and natural resources. As such, the Revised Universal Soil Loss Equation (RUSLE) was implemented (pre-; post-fire) at the Rafina, Attika watershed encompassing the Mati WUI. Fire severity was evaluated based on the Normalized Burn Ratio (NBR). This index was developed utilizing innovative remotely sensed Earth Observation data (Sentinel-2). The high post-fire values indicate the fire's devastating effects on vegetation loss and soil erosion. A critical “update” was also made to the CORINE Land Cover (CLC) v. 2018, by introducing a new land use class namely “Urban Forest”, in order to distinguish the WUI configuration. Post-fire erosion rates are notably higher throughout the study area (4.53–5.98 t ha^−1^ y^−1^), and especially within the WUI zone (3.75–18.58 t ha^−1^ y^−1^), while newly developed and highly vulnerable cites now occupy the greater Mati area. Furthermore, archive satellite data (Landsat-5) revealed how the repeated (historical) wildfires have ultimately impacted vegetation recovery and erosional processes. To our knowledge this is the first time that RUSLE is used to simulate soil erosion at a WUI after a fire event, at least at a Mediterranean basin. The realistic results attest that the model can perform well at such diverse conditions, providing a solid basis for soil loss estimation and identification of high-risk erosion areas.

## Highlights

• RUSLE application to assess impacts of the Mati devastating fire on soil erosion.
• First RUSLE application in a Wildland Urban Interface/ WUI at a Mediterranean area.
• Remote sensing data (Sentinel-2) were utilized to assess fire severity (NBR index)
• Post-fire erosion rates are notably higher, especially within the WUI zone.
• Realistic results attest that RUSLE can perform well at such diverse conditions.

## 1 Introduction

Wildfires often result in great losses to property, damaged infrastructure, destroyed ecosystems (Petropoulos et al., 2011), and loss of human life. According to Sifakis et al. (2011), local and national economies are affected as well, through the destruction of marketable assets. In the European Union (EU), most forest fires (up to 90%) occur in Mediterranean countries (De la Rosa et al., 2008, Athanasakis et al., 2017). The Mediterranean climate and biogeographic characteristics facilitate the outbursts of wildfires and the manifestation of post-fire erosion (Vieira et al., 2018). Fire frequency, extent, and severity have increased in the most recent decades (EC, 2002, EEA, 2007). Furthermore, fire hazard is constantly amplified (Shakesby, 2011, Pausas and Paula, 2012), driven by inadequate forest management practices, the ongoing climate change [i.e. prolonged and drier summers; occurrence of abnormally high temperatures (Mouillot et al., 2002, Karali et al., 2013)], abandonment of cultivated lands that leads to disproportionate biomass (fuel mass) accumulation (Moreira et al., 2001), and human activities such as arsons, campfires etc. (Martınez et al., 2009, Mell et al., 2010).

Wildfires cause partial or complete loss of vegetation cover, disruption of the soil's physical properties, reduced biological activity, increased runoff and unhindered transition downstream; manifestation of flooding events, vulnerability of soil to water erosion, and increased sedimentation rates (Mallinis et al., 2009, Varela et al., 2010, Moody et al., 2013). More specifically, the complete loss of vegetative cover has severe and long-term impacts (obstruction of the role of the canopy; rooting system). Forest tree canopy act as a protective barrier against particle detachment during rainfall events, by intercepting rainfall from directly reaching the ground plane. The rooting system stabilizes and enhances the mechanical strength of the soil, influences its effective hydrological depth and increases flow roughness leading to a reduction in runoff and associated erosion (Efthimiou and Psomiadis, 2018). Overall, an exponential increase of soil loss is induced by the evolving decrease of vegetation cover (Wischmeier, 1975, Gyssels et al., 2005). The impacts on soil properties include organic matter loss, bulk density increase (Neary et al., 2005), porosity; infiltration and water retention capacity decreases (Cerda, 1998, Robichaud, 2000), a reduction in cohesiveness; and a decrease in aggregate stability (Andreu et al., 2001).

The most recognizable post-fire effect drivers include: fire characteristics, the local precipitation attributes, surface topography, type of flora, and local geology. More specifically, fire characteristics involve intensity, duration, and severity (Certini, 2005, Fox et al., 2008). Rainfall attributes include depth, intensity, duration, spatio-temporal variability, and the time span between the fire event and the first rainfall episode (Rulli et al., 2006). In terms of local topography, steep (Yassoglou, 1995) and south aspect (Marques and Mora, 1992) slopes are more sensitive to wildfire impact. The type of flora defines the vegetation recovery rate (Pausas et al., 1999, Vega et al., 2005). Finally, prone to erosion and/ or favouring surface runoff and soil sealing [either by sediment (Neary et al., 1999) or ash (Mallik et al., 1984) particles] bedrocks induce significant post-fire effects as well. In the first post-fire year soil erosion and surface runoff are much more apparent and severe against natural conditions (Rulli et al., 2006) being particularly accelerated during the successive wet season.

The significance of fire impact on a catchment's hydrological regime (Soulis et al., 2012) and soil erosion dynamics (on-; off-site consequences) mandates the accurate simulation and prediction of accelerated post-fire soil loss rates (Gimeno-Garcia et al., 2000, Robichaud and Cerda, 2009). Estimates are crucial for scientists and policy makers, in order to evaluate risk, implement prevention and mitigation measures and design rehabilitation planning. Early erosion studies were based on field observations. Yet, the accuracy of such approximations was counterbalanced by their high cost, time consumption, requirement of specialized expertise, and the overall impracticality in performing large-scale measurements. The modernization of computational tools and the parallel development of Geographical Information Systems (GIS) allowed the generation of several erosion models, of different accuracy and complexity i.e. empirical [e.g. EPM (Gavrilovic, 1962), USLE (Wischmeier and Smith, 1978), RUSLE (Renard et al., 1991) etc.], stochastic [e.g. AGNPS (Young et al., 1987), SWAT (Arnold et al., 1998) etc.] and deterministic [e.g. ANSWERS (Beasley et al., 1980), WEPP (Nearing et al., 1989), KINEROS (Woolhiser et al., 1990), EUROSEM (Morgan et al., 1998) etc.]. These models have evolved into critical decision-making tools, facilitating the problems encountered in conducting field sampling at large spatial scales. The decision on selecting the appropriate type is driven by data availability, local conditions, the physical extent of the study area, and aspired degree of accuracy. Several researchers (Di Piazza et al., 2007, Fernandez et al., 2010, Rulli et al., 2013, Karamesouti et al., 2016) have chosen the empirical RUSLE model (Renard et al., 1991) in order to assess post-fire erosion and land use change implications at Mediterranean areas. Esteves et al., 2012, Karamesouti et al., 2016 have utilized the PESERA (Pan-European Soil Erosion Risk Assessment) (Kirkby et al., 2003) model in predicting the changes in spatial variability of soil erosion following a wildfire event. Other models e.g. Disturbed WEPP (Larsen and McDonald, 2007), EPM (Myronidis and Arabatzis, 2009), MMF (Fernandez et al., 2010), have also been used to evaluate post-fire soil loss rates. RUSLE was chosen for the present study considering its simplicity, ease of use, computational speed, and low demand for input data.

Furthermore, Earth Observation (EO) technology allows the monitoring of landscape dynamics over large areas. Satellite imagery enables the acquisition of cost-effective and time-saving field data for extensive and difficult to access regions, providing moreover continuous measurements (Shanmugapriya et al., 2019, Psomiadis et al., 2019). Therefore, EO multispectral data is also a critical tool of universal applicability for detecting, monitoring, and assessing the damaging impacts of wildfires (Mallinis et al., 2008, Frantzova, 2012) and of post-fire soil erosion and loss potential (Mallinis et al., 2009, Mallinis et al., 2016). Both optical and radar satellite remote sensing have proven to provide significant insight in natural disasters events. Fire severity estimation through remote sensing can be tracked utilizing either optical sensors using vegetation indices or active sensors, especially when using L-band wavelength which is sensitive to temporal changes in vegetation structure (Tanase et al., 2015, Martinis et al., 2017). The European Commission has developed the European Forest Fire Information System (EFFIS) (http://effis.jrc.ec.europa.eu/) to provide a fire risk forecast and a fire danger assessment in EU countries. EFFIS is one of the Copernicus Emergency Services and becomes an essential tool for providing most up-to date information on fire danger in EU, identify the evolution of wildfires and help national authorities to monitor these wildfires (San-Miguel-Ayanz et al., 2013).

Wildland-Urban Interface (WUI) areas comprise “*the transition zones between cities and wildland, where structures and other human development meet undeveloped wildland or vegetative fuels*” (FAO, 2002). Given the highly disastrous impacts of forests fires on such zones (Penman et al., 2014, Alexander et al., 2015), the probability of constant expansion towards fire-prone wildlands especially in the Mediterranean (Theobald and Romme, 2007) and the lack of harmonised transnational forest protection legislation in Europe [each country produces its own policy; buffer zones around urban settlements/wildlands range from 50 to 200 m/100–400 m, respectively (Modugno et al., 2016)], these areas need to be prioritized by developing fire control/ management plans to minimize the associated risk (Hammer et al., 2009, Massada et al., 2009, Mitsopoulos et al., 2015).

The purpose of the study is to assess the impacts of the devastating fire of July 2018 at Mati, Attika on soil erosion based upon its size, intensity, socio-economic impact, and environmental consequences. To that end, RUSLE was applied (pre-; post-fire) to the Rafina watershed, including the WUI zone of the Mati settlement. To our knowledge this is the first time that RUSLE is used to model soil erosion at a WUI after a fire event, at least in a Mediterranean region. Apart from the implementation of RUSLE at the WUI zone, the novelty of the study also involves the analytical calculation of the R-factor based on the EI_30_ index, the analytical calculation of the K-factor based on field samples, the “upgrade” of the CORINE (Coordination of Information on the Environment) Land Cover (CLC) v. 2018 in order to describe the specific configuration of the WUI, and the utilization of remote sensing techniques in order to calculate the C-factor and assess the severity of the fire.

## 2 Materials and methods

**Fig. 1:**
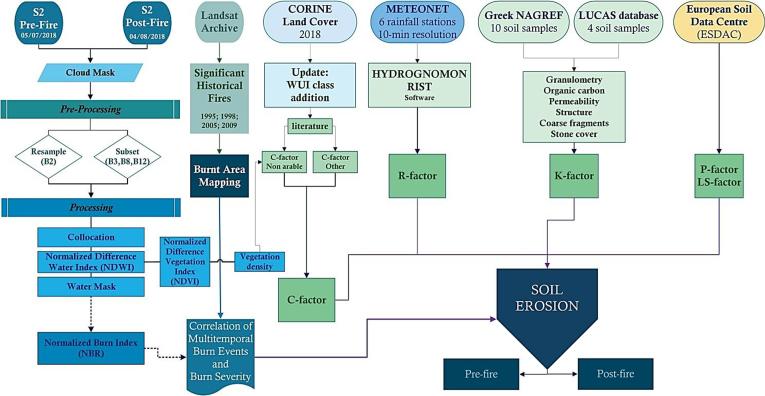
Methodology flow chart.

The flowchart (Fig. 1) presents schematically the methodology followed in this study.

### 2.1 Study area

**Fig. 2:**
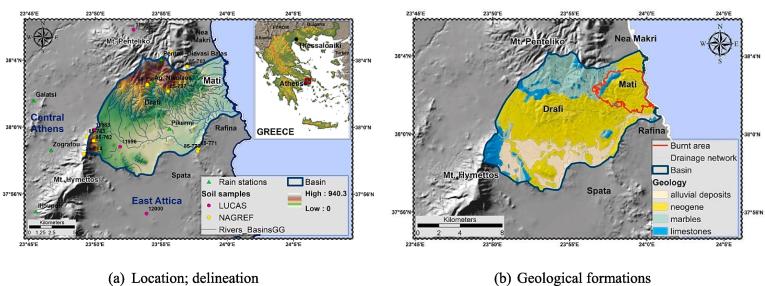
Study area.

The study area, namely Rafina catchment, is located in Attica, Greece, between 37^o^56′ to 38^o^05′ N and 23^o^45′ to 24^o^00′ E. The study site is located approximately 25 km northeast of Athens [Fig. 2(a)], occupying an area of 135.32 km^2^. Dominant topographic features include the Hymettus and Penteli mountains, being bound by shallow basins oriented in a northwest–southeast and northeast–southwest direction (Mettos et al., 2000). The elevation ranges between 0 and 940 m, having a mean value of 227.8 m; steep slopes characterize the northern regions of the catchment while the relief is milder at the eastern part towards its outlet. The hydrographic network is relatively dense, presenting a more complex form to the northern and eastern parts of the catchment. The drainage pattern is of a dendritic type. The broader Eastern Attika experiences a Mediterranean climate type, featuring hot and dry summers and mild and wet winters. The bedrock of the study area is mainly comprised of Neogene formations of Upper Pliocene–Lower Pleistocene, Quaternary and Pleistocene alluvial deposits, limestones, and marbles [Fig. 2(b)] (Alexakis, 2008). The encompassed area of the Mati settlement that was damaged by the wildfire occupies 14.49 km^2^ of the basin [Fig. 2(b)].

#### 2.1.1 The devastating fire of 2018

**Fig. 3:**
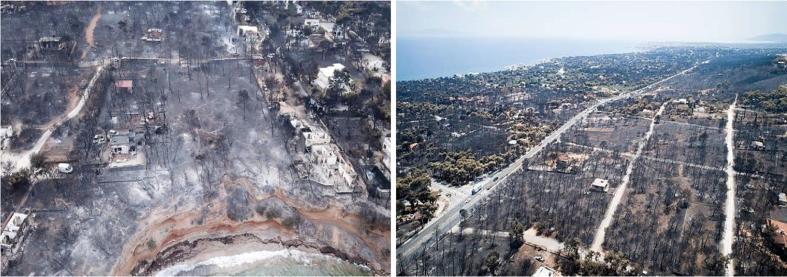
Impacts of the devastating fire of 2018 at Mati, Attika.

The crown fire of 2018 at Mati, Attika has been referred to as the deadliest natural disaster in the history of the Modern Greek state. The fire broke out on July 23 at the forest surroundings of the Ntaou region on Penteli Mountain, eastern Attica, approximately 20 km northeast of Athens and 5 km off the Eastern Attica coast. It was initially directed towards the Dionysos area at normal rate, burning low vegetation. Due to the extreme weather conditions, i.e. high temperatures (nearly 40 °C), low relative humidity (around 19%), locally gale force winds [velocity of up to 124 km h^−1^ or 12 Beaufort (highest grade on the respective scale), (http://meteo.gr /UploadedFiles/articlePhotos/JUL18/MaxGusts_Attica_23072018.png) (accessed 24 January 2018)], it spread swiftly to the east towards Rafina, directly impacting several local settlements. The fire reached the settlement of Mati (in almost 30–40 min), where it finally stopped, meeting the coastline; the fire front was nearly 1 km wide (Fig. 3). According to EFFIS assessments for 23 July 2018, the fire danger conditions in the Attika region (around Athens) were of very high to extreme levels, specifically in the locations of Rafina where the fire occurred (EU Science hub news, 2018).

Overall, the adverse weather conditions, the regional morphology and microclimate, the town configuration and the differences in fuels created ideal conditions for rapid fire spreading. More specifically, gale force west‐northwest winds over Attica with gusts often exceeding 100–120 km h^−1^ allowed for minimum response time. The latter act as downslope winds in the eastern regions of Attica resulting in significant temperature rise and low humidity. Furthermore, fuel differences (see Section 2.1.2; type and amount of vegetation due to previous fires) could have led to alterations in fire behavior and the rate the front spread eastwards towards Mati. Additionally, the poor spatial planning (houses built near or even within pine forest at random density; incomplete road network with narrow streets and dead ends; not a single street was designed to provide direct access to the sea; paths leading to the sea were blocked by fences; rocky and steep seashores; lack of public gathering places etc.) and the lack of firewalls (Marathon Avenue, given its 15 m width could have served as such, yet the unprecedented severity of the fire and the inadequate coordination measures led to its overcome) resulted in the entrapment of a significant number of people.

102 human lives were lost, hundreds of square kilometres of Aleppo pine (Pinus Halepensis) forest (locally mixed with Cypress trees and shrubby vegetation, especially within the WUI zone); cultivated parcels and urban greenery were incinerated, >1,500 buildings were destroyed or suffered damages and many vehicles (3 0 5) were ruined. Local residencies were evacuated and abandoned, with numerous people ending up homeless, struggling with recovery or rebuilding efforts up to this day. Severe damages were caused to the electricity, telecommunication and water supply network as well. Apart from the direct losses, long term impacts involve health issues from impaired air quality due to the wildfire smoke and heavy metals melting, and a wide range of financial impacts such as a downturn in tourism, business and recreation revenue (ecological damage harmed the natural resource base from which the local community derived economic activity and employment), insurance costs and public funding for disaster assistance, legal costs, and impacts on property and housing values within or near the fire perimeter due to proximity with burned landscapes.

#### 2.1.2 Historical fires in the region

**Fig. 4:**
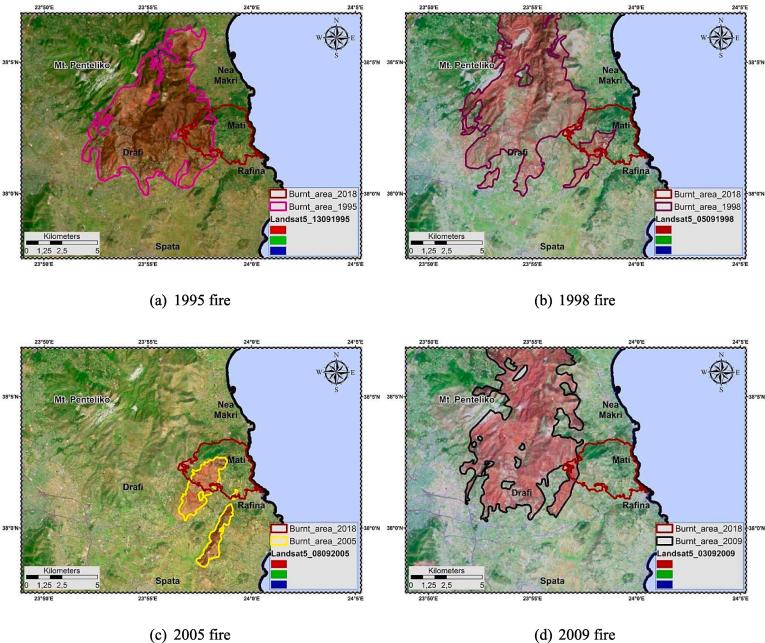
Historical fires in eastern Attika; Burned areas delineation using Landsat-5 archive satellite images.

Apart from the fire event of 2018, other wildfires had previously occurred within the broader study area (Fig. 4). On 21 July 1995 a large fire broke out on Penteli Mountain, Attica, in a thick pine forest (Xanthopoulos, 2002). The fire burned about 251 km^2^ of land. Almost 105 buildings were heavily damaged or destroyed, yet no human lives were lost (Xanthopoulos, 1996). On 2 August 1998 a fire started at the northeast of Athens. The fire reached the 1995 burned area, re-incinerating any regenerated vegetation, while entered “deeper” into the settlements located at its perimeter (Xanthopoulos, 2006). It also affected unburned forest at the Penteli village outskirts. Hundreds of houses and public buildings were destroyed or seriously damaged; one civilian lost his life (Xanthopoulos, 2006). On 28 July 2005 another fire started from Rafina, eastern Attika. Its two fronts burnt 4 km^2^ of land resulting in damages to numerous buildings (Xanthopoulos, 2006). On 21 August 2009 a wildfire broke out in eastern Attica where it burned a total of 850 km^2^ of land, mainly pine forest. The fire extended throughout the entire north-eastern Attica. Around 72 houses were damaged, and several local communities were heavily affected. This fire is considered as one of the greatest, ever known in the prefecture of Attica.

### 2.2 Data and measurements

#### 2.2.1 Earth Observation (EO) data

Across almost five decades, the NASA/USGS's program of Landsat satellites offers the longest continuous space-based record of Earth's surface. Landsat-5 (L5) was the fifth satellite of the Landsat mission, equipped with Thematic Mapper (TM) that operates on six multispectral bands (30 m pixel size) and a thermal band (pixel size 60 m). Moreover, the Copernicus program of the European Space Agency (ESA) provides innovative and continuous satellite data. Sentinel-2 (S2) is an optical satellite, which delivers very high spatial analysis imagery having a temporal resolution of five days. S2 is equipped with the MSI (Multi-Spectral Imager) sensor that operates on 13 different bands at a spatial resolution of 10 (4 bands), 20 (6 bands) and 60 (3 bands) m. Its data have significant usage in researches dealing with land cover monitoring and change detection (Psomiadis et al., 2019).

Several data sets were acquired for this study. S2 database is available free of charge via the ESA portal (https://scihub.copernicus.eu/). The available S2 products contained a processing Level-2A, geometrically and atmospherically corrected. Two S2 images (pre- and post-fire) were acquired for the burn scar delimiting and impact assessment (Table 1). Moreover, four L5 TM Level 1 T images were obtained free of charge through the USGS Earth Explorer (EE) tool (https://earthexplorer.usgs.gov/), proven valuable in delineating the most severe historical fires that occurred within the area. The images were geometrically corrected having a standard terrain correction applied (Table 1). The digital image processing and spatial analysis were accomplished utilizing ENVI (5.5, Harris Geospatial Solutions, USA), ArcGIS (10.5.1, Environmental Systems Research Institute, Redlands, CA, USA) and SNAP (6.0, European Space Agency) software.

**Table 1:** Satellite images datasets.

Satellite System	Instrument	Image code/source	Acquisition	Use
Sentinel-2	MSI	S2B_MSIL2A_20180705T091019_N02 08_R050_T34SGH_20180705T133603	05/07/2018	Burn Scar delineation & Burn Severity extraction
		S2B_MSIL2A_20180804T090549_N02 08_R050_T34SGH_20180804T142040	04/08/2018	

Landsat 5	TM	LT05_L1TP_182034_19950913_20180210_01_T1	13/09/1995	Historical Burn Scar delineation
		LT05_L1TP_182034_19980905_20180727_01_T1	05/09/1998	
		LT05_L1TP_182034_20050908_20180126_01_T1	08/09/2005	
		LT05_L1TP_182034_20090903_20161021_01_T1	03/09/2009	

#### 2.2.2 Geospatial data

The Hellenic Military Geographical Service (HMGS) provided the topographic map (sheet “Kifissia”, scale 1:50,000), used to extract different types of info-layers: geomorphology, rivers, roads, railway tracks etc. A detailed DEM with 5-m spatial resolution and 0.5-m in geolocation, deriving from topographical maps (scale 1:5,000, contour interval 4 m) of HMGS, was also used for the purposes of the study.

**Fig. 5:**
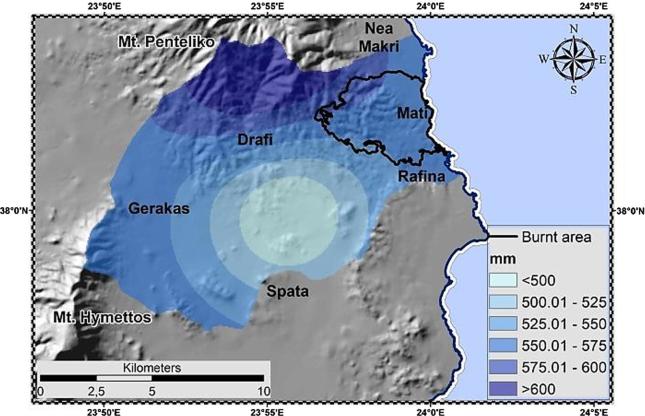
Mean precipitation of the study area.

Precipitation measurements were derived from the METEONET network. METEONET includes over 10 fully automatic telemetric stations located in the wider Athens area; it has been developed and maintained by the National Technical University of Athens (NTUA) and more specifically by the Centre for Hydrology and Informatics (CHI) (http://www.chi.civil.ntua.gr/). The locations of the network's stations [Fig. 2(a), Table 2] were selected to adequately cover the area of interest and meet strict monitoring criteria set by WMO (1996) (Grammatikogiannis et al., 2005). The meteorological stations' data loggers, record data in 10 min intervals (http://hoa.ntua.gr/) (accessed 25 January 2019). The rain map of the study area is presented in Fig. 5. Precipitation was spatially distributed based on its mean annual values (Table 2), utilizing the co-kriging interpolation method and using as a covariate the basin elevation.

**Table 2:** Precipitation stations.

Station	Latitude	Longitude	Elevation (m)	Interval (min)	Records^2^	Period (y)	P^1^ (mm)	R^1^ (MJ mm ha-^1^h−^1^ y−^1^)
Agios Nikolaos	491695.78	4210665.46	383	10	19/10/2003–13/11/2015	12	616.77	999.5
Galatsi	478563.95	4208803.49	176		15/06/2005–16/02/2018	13	412.03	702.2
Ilioupoli	478837.90	4196512.17	206		20/05/2005–17/07/2017	12	460.67	1047.1
Penteli (Diavasi Balas)	492698.31	4213335.07	630		10/12/2003–15/11/2015	12	665.17	1683.33
Pikermi	493583.15	4205666.35	133		21/12/2005–16/02/2018	13	452.89	886.02
Zografou	480493.49	4203306.04	181		05/08/2005–25/04/2018	13	576.98	1252.29

1 Total values represent the sum of monthly averages.

2 Relatively different periods; due to their minor deviations they are considered consistent.

The definition of pedological properties was made considering fourteen (14) soil samples [Fig. 2(a)], provided by the Greek National Agricultural Research Foundation (NAGREF) and the Land Use/Cover Area frame Survey (LUCAS) database (Orgiazzi et al., 2018). The samples refer only to the surface layer (0–30 cm depth), delivering information of several attributes per sampling site, namely soil type, parent material, granulometry, organic matter content, nutrient content, surface coarse fragments abundance, subsurface stoniness, etc.

**Fig. 6:**
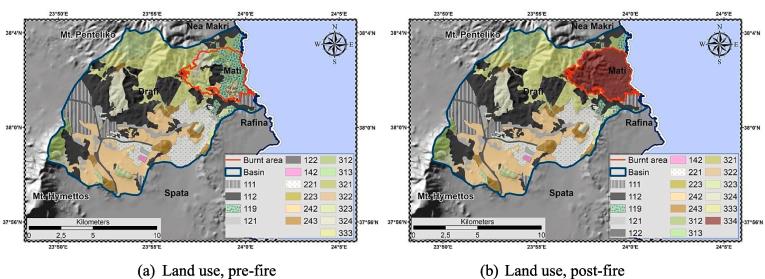
Land use, pre-; post-fire delineation.

**Fig. 7:**
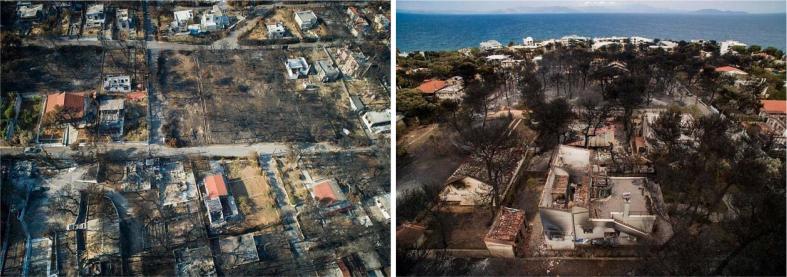
Indicative city layout/configuration of Mati, Attika.

The CLC catalogue was used to illustrate land use. CLC is a static, classified, continental-scale land cover map, utilizing a Minimum Mapping Unit (MMU) of 25 ha for areal phenomena. In the present study the latest CLC2018 (CLC, 2018) was managed for pre-fire and post-fire delineation (Fig. 6). Considering the CLC limitations [by using a 25 ha MMU, higher resolution parcels may be missed; biased identification of WUIs at small-scale delineations, particularly in urban surrounding regions (Diaz-Pacheco and Gutierrez, 2014)] and the Mati settlement spatial planning [developed near or even within pine forest, forming a WUI (Fig. 7)], a critical “update” was made by introducing a new class named “Urban Forest” in order to distinguish such configuration (Table 3).

**Table 3:** CLC 2018 land use classification; pre- and post-fire delineation (shaded classes show significant changes).

		Pre-fire		Post-fire	
Code	Land use	km^2^	%	km^2^	%
111	Continuous urban fabric	9.96	7.36	9.80	7.24
112	Discontinuous urban fabric	29.97	22.15	28.06	20.73
119^1^	Urban forest	7.22	5.34	2.00	1.48
121	Industrial or commercial units	4.11	3.04	4.11	3.04
122	Roads and Rails	1.91	1.41	1.91	1.41
142	Sport and leisure facilities	0.26	0.19	0.26	0.19
221	Vineyards	12.26	9.06	12.24	9.05
223	Olive groves	0.22	0.16	0.15	0.11
242	Complex cultivation patterns	21.88	16.17	20.89	15.44
243	Land principally occupied by agriculture, with significant areas of natural vegetation	4.93	3.65	4.93	3.65
312	Coniferous forest	5.40	3.99	3.84	2.83
313	Mixed forest	0.40	0.30	0.24	0.18
321	Natural grassland	0.09	0.06	0.09	0.06
322	Moors and heathlands	0.13	0.10	0.07	0.05
323	Sclerophyllous vegetation	6.55	4.84	6.55	4.84
324	Transitional woodland/shrub	7.45	5.51	5.78	4.27
333	Sparsely vegetated areas	22.57	16.68	19.93	14.73
334	Burnt areas	0.00	0.00	14.49	10.70
	Σ	135.32	100.00	135.32	100.00

1 new class, developed to describe the specific land use characteristics of the study area.

### 2.3 The Revised Universal soil loss Equation (RUSLE)

Renard et al. (1991) developed the Revised Universal Soil Loss Equation (RUSLE). RUSLE is an empirical soil erosion model that estimates gross (sheet; rill) erosion, based on five parameters, namely the rainfall erosivity (R-factor); soil erodibility (K-factor); topographic (LS-factor); cover management (C-factor); and conservation practice (P-factor) factors. Mean annual soil loss (A, t ha^−1^ y^−1^) is estimated according to Eq. (1).

(1) A=R×K×LS×C×P

The R-factor (MJ mm ha^–1^h^−1^ y^–1^) is the model's climatic component, accounting for the effect of precipitation on soil loss. The rainfall erosivity index *EI_30_* (MJ mm ha^–1^h^−1^) (Wischmeier, 1959) describes the erosive action of an individual rain event *k*. *EI_30_* is estimated by multiplying the rainfall kinetic energy *E* to its maximum 30-min intensity *I_30_* (Brown and Foster, 1987) (Eq. (2)). *E* is calculated per time interval *r*, as the sum of the rainfall volume *v_r_* (mm) times its unit energy *e_r_* (MJ ha^−1^ mm^−1^) products (Brown and Foster, 1987), with the *e_r_* being a function of rainfall intensity *i_r_* (mm h^−1^) (Eq. (3)). For any given period, *n,* the annual rainfall erosivity index derives by summing the *EI_30_* values of all erosive events *mj* that occurred within a year's time *j* (Renard et al., 1996) (Eq. (4)).

(2) $$EI_{30}=\left(\sum_{r=1}^{0}e_{r}v_{r}\right)I_{30}$$

(3) $$e_{r}=0.29\left[1-0.72\exp\left(-0.05i_{r}\right)\right]$$

(4) $$R=\frac{1}{n}\sum_{j=1}^{n}\sum_{k=1}^{\mathrm{mj}}{\left(EI_{30}\right)}_{k}$$

The K-factor (t ha h ha^−1^ MJ^−1^ mm^−1^) is the model's pedological component, describing the susceptibility of soil against the actions of erosive rainfall and overland flow. The erodibility of the soil is estimated as a function of its characteristics, namely the grain size parameter *M*, the organic matter content *a* (%), the structure *b* and permeability *c* (Eq. (5)). The calculation of *M* is based on the “silt + very fine sand” fraction *P_s_* (%) and clay fraction *P_c_* (%) contents (Eq. (6)).

(5) $$K=\left[\left(2.1M^{1.14}\left(10^{-4}\right)\left(12-a\right)+3.25\left(b-2\right)+2.5\left(c-3\right)\right)/100\right]0.1317$$

(6) $$M=P_{s}\left(100-P_{c}\right)$$

The LS-factor (dimensionless) is the model's topographic component. It is comprised by the slope length *L* (Wischmeier and Smith, 1978) (Eq. (7); fraction) and steepness *S* (McCool et al., 1987, McCool et al., 1989) (Eq. (7); bracket) individual factors, describing their combined effect on erosion. The topographic factor is estimated as function of the slope's attributes i.e. horizontal projection of its length *λ* (ft) raised to a power of *m* (classified; calculated based on the rill to inter-rill erosion ratio *β*), angle *θ* and steepness *S* (%).

(7) $$\mathrm{LS}={\left(\frac{\lambda }{72.6}\right)}^{m}\left\{\begin{array}{c} 10.8\sin\theta +0.03,S<9\%\\ 16.8\sin\theta -0.50,S⩾9\%\\ 3.0{\left(\sin\theta \right)}^{0.8}+0.56,\lambda <15ft \end{array}\right)$$

The C- and P- factors (dimensionless) account for the protective effect of vegetation and conservation measures (e.g. tillage practices in arable lands) against soil erosion, respectively.

#### 2.3.1 Limitations of the RUSLE model

The RUSLE model describes the complex and highly non-linear soil erosion “mechanism” in a simplified manner (Kirkby, 1980), through the linear multiplication of completely different individual parameters; drivers (rainfall, soil characteristics, topography, vegetation cover, and erosion control practices), while important processes like sediment transport; routing (within the hydrographic network), deposition etc. are not considered. Moreover, given the fact that the model disregards such processes, the transcendence from local soil loss risk assessment (gross erosion) to the estimation of sediment yield at the outlet of a given catchment requires the use of external coefficients like the Sediment Delivery Ratio. Also, RUSLE can only simulate sheet and rill (inter-rill) erosion, displaying inability to model gully and channel erosion. Additionally, its applicability on large spatial scales (e.g. catchment, nationwide, continental) where non-uniform climatic, land cover, soil type etc. conditions occur is controversial. Finally, its prediction accuracy at individual flood events – given the important influence of unforeseen random fluctuations – is low (significant deviation from the average values). Contrary, at longer time scales such accuracy is notably improved, since the variation is smoothed to the long-term average of the soil loss value (Efthimiou et al., 2014).

However, despite these limitations, the model can provide a solid basis in terms of soil erosion assessment and identification of high-risk soil loss areas, either as a rough initial; preparatory approach (at regions characterized by availability of detailed input data that will facilitate the subsequent application of more comprehensive; process-based models) or as the basic methodological approximation (at regions characterized by scarcity of input data of high spatio-temporal resolution).

### 2.4 Earth Observation data processing

#### 2.4.1 The Normalized difference vegetation index (NDVI)

The Normalized Difference Vegetation Index (NDVI) expresses the difference between wavelength reflectance in two portions of the electromagnetic spectrum, the visible Red and the near-infrared (NIR) (Eq. (8)) (Rouse et al., 1973), corresponding to the relative vegetation properties i.e. low reflectance in the Red and high reflectance in the NIR. The index acquires values in the range − 1 to + 1. In the lack of vegetation (e.g. bare soil, rock, urban areas) NDVI acquires low positive (0–0.2) or negative values (water bodies). Contrary, in the presence of dense vegetation (e.g. grassland, forests etc.) the index acquires values from 0.2^−^1 (Sader and Winne, 1992, Lillesand et al., 2004).

(8) $$\mathrm{NDVI}=\frac{\mathrm{NI}R_{B8}-{\mathrm{RED}}_{B4}}{\mathrm{NI}R_{B8}+{\mathrm{RED}}_{B4}}$$

#### 2.4.2 The Normalized Burn Ratio (NBR)

The Normalized Burn Ratio (NBR) is used for highlighting burned areas, having the ability to indicate burn severity. It serves as a proxy of fire characteristics, towards assessing fire impact on vegetation and soil attributes and by extension on soil erosion. Healthy vegetation displays very high reflectance in the NIR band, and low reflectance in the shortwave infrared (SWIR) one. Burned areas on the other hand display relatively low reflectance in the NIR portion of the spectrum, while high in the SWIR one. High NBR values indicate healthy vegetation in general, and low, bare ground and recently burned areas (López-Garcia and Caselles, 1991, Keeley, 2009). The NBR formula utilizes the NIR (B8) and infrared (B12) satellite bands of S2 (Eq. (9)).

(9) $$\mathrm{NBR}=\frac{\mathrm{NI}R_{B8}-SWIR_{B12}}{\mathrm{NI}R_{B8}+SWIR_{B12}}$$

where, B4; B8 are the respective satellite bands of S2.

## 3 Results and discussion

### 3.1 NBR development

The S2 products conveniently contain vector cloud and cirrus masks, which are created as a product of the atmospheric correction. Towards estimating the NBR index, a cloud mask was initially applied. Subsequently, resample (using band 2), subset (selecting bands 3, 8 and 12 and cloud mask) and Band Math [calculating Normalized Difference Water Index (NDWI) (Eq. (10)) and the NBR (Eq. (9))] processes were implemented, utilizing the Graph Builder tool of the SNAP software. Water bodies can display similar NBR difference in certain circumstances; therefore, it is necessary to mask them out by creating a single combined water mask. To detect the presence of water bodies, the NDWI was calculated utilizing near-infrared band 8 and the Green band 3 (Gao, 1996).

(10) $$\mathrm{NDWI}=\frac{\mathrm{Gree}n_{B3}-NIR_{B8}}{\mathrm{Gree}n_{B3}+NIR_{B8}}$$

**Fig. 8:**
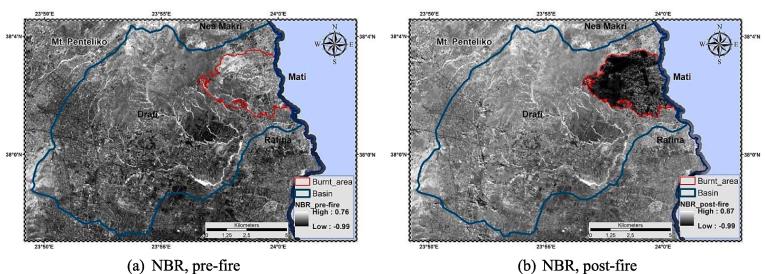
Normalized Burn Ratio (NBR), pre-; post-fire delineation.

Subsequently, NBR was estimated before and after the wildfire using Eq. (9) (Fig. 8).

### 3.2 RUSLE application

RUSLE was implemented in a GIS environment, using the ArcGIS 10.5.1 platform. Each parameter acquired the form of a digital map, and soil loss was estimated on a cell-by-cell basis.

The availability of detailed temporal resolution (10-min) precipitation measurements allowed the analytical estimation of the R-factor, based on *EI_30_*. Before any calculations were made, the rainfall values were aggregated to the desirable/theoretical 30-min temporal resolution. This is because direct calculation of the R-factor from lowest to highest precipitation timescale leads to gradual underestimation of its value (Williams and Sheridan, 1991, Yin et al., 2007). The aggregation and the infilling of the time series gaps were made, using the HYDROGNOMON v.4.1.0.26 software, an open source software developed by the NTUA (Kozanis and Markonis, 2009).

The R-factor was estimated (Table 2) using the RIST (Rainfall Intensity Summarization Tool) platform (USDA, 2014). Low intensity rain events i.e. with depth less than 12.7 mm were excluded from the energy and intensity calculations, since they have a minor effect on *EI_30_*. The R-factor spatial distribution utilized the kriging interpolation methodology. Overall, the most erosive rainfall events are characterized by high R-factor values (Fig. 5). Mean R-factor was calculated as 1174.81 MJ mm ha^−1^h^−1^ y^−1^having variability range of 1126.10–1260.70 MJ mm ha^−1^h^−1^ y^−1^.

The availability of soil samples allowed the analytical estimation of the K-factor, based on the nomograph developed by Wischmeier et al. (1971). The process involved the initial categorization of the samples according to their granulometric composition (SSDS, 2017), their clustering into greater textural groups and the assignment of the structure *b* and permeability *c* (Rawls et al., 1982) indices codes. Given the granulometry classification, a theoretical value (Clapp and Hornberger, 1978) of Saturated Hydraulic Conductivity (SHC) (*K_f_*, mm h^−1^, fine soil fraction less than 2 mm) was ascribed to each sample. Since soil permeability is reduced by the presence of subsurface coarse fragments, their effect was considered by recalculating SHC (*K_b_*, mm h^−1^) using Eq. (11) (Brakensiek et al., 1986). If *K_b_* is assigned to a different SHC class (Neitsch et al., 2005) than *K_f_*, the permeability index value changes accordingly.

(11) $$K_{b}=K_{f}\left(1-R_{w}\right)$$

where, *R_w_* is the ratio of coarse fragments > 2 mm.

Additionally, for samples with organic matter content higher than 4%, an upper limit equal to this value was imposed according to the nomograph restrictions. Since surface stoniness/stone cover (*R_c_*, %) reduces soil erodibility i.e. sediment yield (Panagos et al., 2014), its effect was also incorporated by using Eq. (12) (Poesen et al., 1994).

(12) $$\mathrm{St}=\left\{\begin{array}{c} 1,R_{c}<10\%\\ e^{-0.04\left(R_{c}-10\right)},10\%⩽R_{c}<100\% \end{array}\right)$$

where, St is the correction factor.

The final K-factor value *K_St_*, including all limitations, was calculated by multiplying the parameter's delimited value with the correction coefficient (Eq. (13)) (Table 4).

(13) $$K_{\mathrm{St}}=St\times K$$

**Table 4:** K factor analytical estimation.

Code^1^	Class^2^	Group^3^	Structure^4^	b^5^	Permeability^6^	a^7^	Coarse^8^	c^9^	Gravels^10^	St^11^	K_St_^12^
702	SCL	MF	MCG	3	ML	4.00	27.5	4	60	0.14	0.004
703	SCL	MF	MCG	3	ML	3.60	10	4	10	1.00	0.022
736	SCL	MF	MCG	3	ML	4.00	10	4	60	0.14	0.003
737	SCL	MF	MCG	3	ML	4.00	10	4	10	1.00	0.022
761	SCL	MF	MCG	3	ML	2.81	10	4	60	0.14	0.003
762	CL	MF	MCG	3	ML	3.30	3.5	4	60	0.14	0.004
763	SL	MC	MCG	3	MF	4.00	3.5	2	60	0.14	0.003
764	C	F	FG	2	VS	4.00	27.5	6	60	0.14	0.005
770	SCL	MF	MCG	3	ML	4.00	10	4	27.5	0.50	0.015
771	SCL	MF	MCG	3	ML	3.94	10	4	27.5	0.50	0.011
11,983	SL	MC	MCG	3	MF	0.17	18	2	37.5	0.33	0.006
11,996	CL	MF	MCG	3	ML	1.87	12	4	5	1.00	0.026
11,998	SL	MC	MCG	3	MF	3.58	13	2	17.5	0.74	0.008
12,000	SL	MC	MCG	3	MF	2.82	5	2	5	1.00	0.006

1 Overseer: NAGREF (702–771 samples), LUCAS (11983–12000 samples).

2 SCL: Sandy Clay Loam, CL: Clay Loam, SL: Sandy Loam, C: Clay.

3 MF: Moderate Fine, MC: Moderate Coarse, F: Fine.

4 MCG: Medium or Coarse Granular, FG: Fine Granular.

5 Soil structure index: 2 (Fine Granular), 3 (Medium or Coarse Granular).

6 ML: Moderate Low, VS: Very Slow, MF: Moderate Fast.

7 OM content (%) – upper 4% limit exceedance marked in grey background.

8 In %.

9 Soil permeability index: 2 (Moderate Fast), 4 (Moderate Low), 6 (Very Slow)

10 In %.

11 Correction factor – values marked in grey background for Rc (stone cover percentage) lower than 10%.

12 K_St_ factor – corrected for OM content and coarse fragments restrictions, in t ha h ha^−1^ MJ^−1^ mm^−1^.

**Fig. 9:**
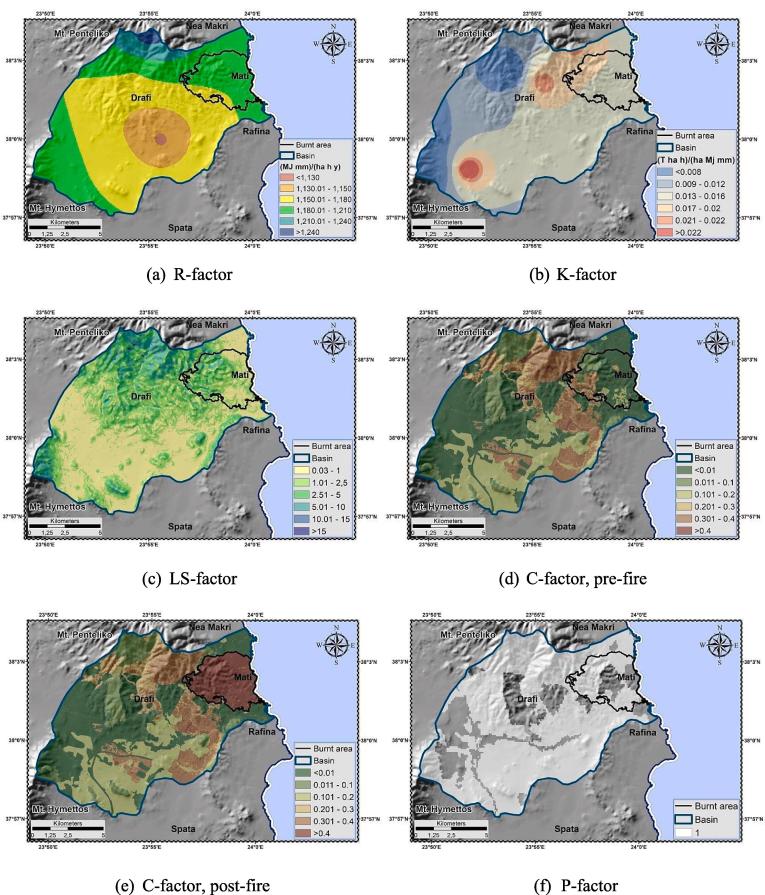
RUSLE factors.

The K-factor spatial distribution utilized the Inverse Distance Weighing (IDW) method (Angulo-Martinez et al., 2009, Panagos et al., 2012). Overall, high K-factor values characterize the most erodible soils (Fig. 9). Mean K-factor was calculated as 0.0127 t h MJ^−1^ mm^−1^ having variability range of 0.003–0.026 t h MJ^−1^ mm^−1^.

The EU LS-factor (Panagos et al., 2015a) and P-factor (Panagos et al., 2015b) datasets, made available by the European Soil Data Centre (ESDAC), were used for estimating the topographic and support practice factors, respectively. Overall, the highest LS-factor/P-factor values characterize areas of strong relief/absence of prevention measures. Mean LS-factor was calculated as 1.99 having variability range of 0.03–47.58. P-factor acquired a unit value, accounting for the lack of recorded protection measures in the region (Fig. 9).

**Fig. 10:**
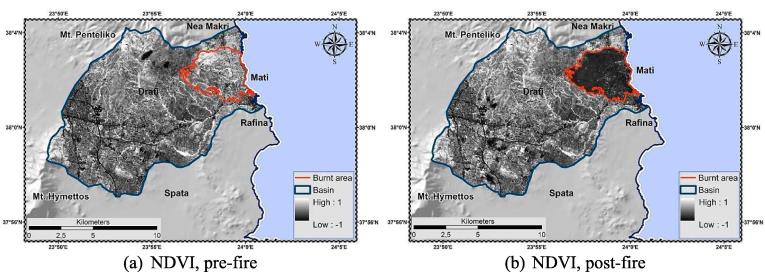
NDVI, pre-; post-fire delineation.

The CLC (2018) delineation was the base-map for the cover management factor estimation. Each class was initially assigned with an empirical C-factor value (Wischmeier and Smith, 1978, Panagos et al., 2015c) (Table 5). Subsequently, in non-arable lands C-factor was re-calculated following the methodology proposed by Panagos et al. (2015c). The latter accounts for the effect of vegetation density variations (Lu et al., 2004) in the estimation of C-factor, considering the combined impact of biomass (above-; below-ground) and environmental conditions (Smets et al., 2008). According to such approach, a range of C-factor values (C_Landuse_) was ascribed to each non-arable CLC class, while proxy vegetation layer [e.g. Fraction of Vegetation Cover, F_cover_ (https://land.copernicus.eu/global/products/fcover) (accessed 25 January 2019)] was used for the quantification of the vegetation cover impact in the estimation process (Eq. (14)). In the present study, the proposed F_cover_ dataset was replaced by the NDVI (normalized in the range 0–1), serving as proxy pre-; post-fire vegetation cover layer (Fig. 10) (instantaneous information, representative of the time of the satellite images acquisition). NDVI was estimated based on Eq. (8). Low C-factor values indicate strong protection against erosion, met at areas of high vegetation cover (Fig. 9). The minimum C-factor value is acquired when the NDVI reaches the unit (soil fully covered by vegetation).

(14) $$C_{\mathrm{NONarable}}=Min\left({C_{\mathrm{landus}}}_{e}\right)+Range\left(C_{\mathrm{landuse}}\right)\times \left(1-NDVI\right)$$

**Table 5:** C-factor values, pre-, post-fire delineation.

					Pre-fire		Post-fire	
Code^1^	C^2^	Range (C_Landuse_)	Range^3^	Min (C_Landuse_)	NDVI	C_w_^4^	NDVI	C_w_^4^
111	0.0001	–	–	0.0001	–	0.0001	–	0.0001
112	0.001	–	–	0.001	–	0.001	–	0.001
119	0.0005	–	–	0.0005	–	0.0005	–	0.0005
121	0.001	–	–	0.001	–	0.001	–	0.001
122	0.0001	–	–	0.0001	–	0.0001	–	0.0001
142	0.005	–	–	0.005	–	0.005	–	0.005
221	0.25	0.15–0.45	0.3	0.15	0.32	0.35	0.32	0.35
223	0.1	0.1–0.3	0.2	0.1	0.32	0.24	0.31	0.24
242	0.18	0.07–0.2	0.13	0.07	0.32	0.16	0.31	0.16
243	0.07	0.05–0.2	0.15	0.05	0.36	0.15	0.36	0.15
312	0.002	0.0001–0.003	0.0029	0.0001	0.52	0.0015	0.50	0.0015
313	0.001	0.0001–0.003	0.0029	0.0001	0.62	0.0012	0.57	0.0013
321	0.05	0.01–0.08	0.07	0.01	0.26	0.06	0.26	0.06
322	0.17	0.01–0.1	0.09	0.01	0.56	0.05	0.50	0.05
323	0.03	0.01–0.1	0.09	0.01	0.51	0.05	0.48	0.06
324	0.02	0.003–0.05	0.047	0.003	0.45	0.03	0.43	0.03
333	0.45	0.1–0.45	0.35	0.1	0.40	0.31	0.36	0.32
334	0.55	0.1–0.55	0.45	0.1			0.16	0.48

1 221–334: Non-arable.

2 initial C-factor values.

3 the result of maximum–minimum C_Landuse_ values.

4 weighted C-factor values for non-arable lands (Panagos et al., 2015c).

### 3.3 Pre-fire analysis

**Fig. 11:**
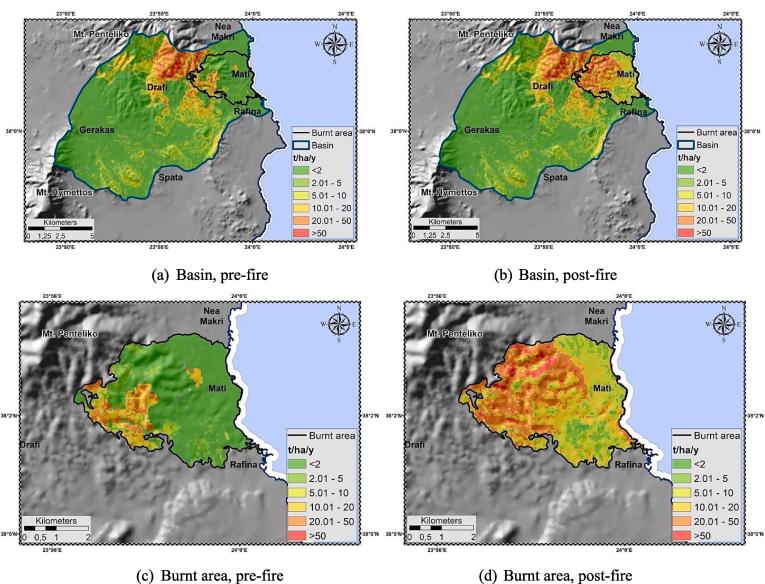
Soil loss, pre-; post-fire analysis.

Analysis comprised two stages, one before the fire and one after it. The pre-fire application of RUSLE yielded mean annual soil loss of 4.53 t ha^−1^ y^−1^, having variability range of 3.44–149.02 t ha^−1^ y^−1^. These values are in accordance with the ones estimated by Panagos et al. (2015d) for the Greek territory, i.e. 4.13 t ha^−1^ y^−1^. The most erosion-prone areas (coloured red) are situated at the north of the basin [Fig. 11(a)], in the highest altitudinal zone. The latter occupy a notable part of the study area, yet with significantly less expanse against the low-risk ones. Regarding the aspect, highly susceptible to erosion areas are cited at slopes facing the south (Marques and Mora, 1992). Focusing on the (imminent) burnt area of the broader Mati settlement, RUSLE implementation yielded mean annual soil loss of 3.75 t ha^−1^ y^−1^, having variability range of 0.00–78.61 t ha^−1^ y^−1^. High-risk areas (red colour) occupy the western part of the settlement [Fig. 11(c)] partially coinciding with the ones of the antecedent watershed. The model showed large variations in erosion rates at the study site (basin, WUI). Notably high values of soil loss, for a few sites (respectively), had a disproportionate effect on its mean value [Fig. 11(a), (c)].

**Fig. 12:**
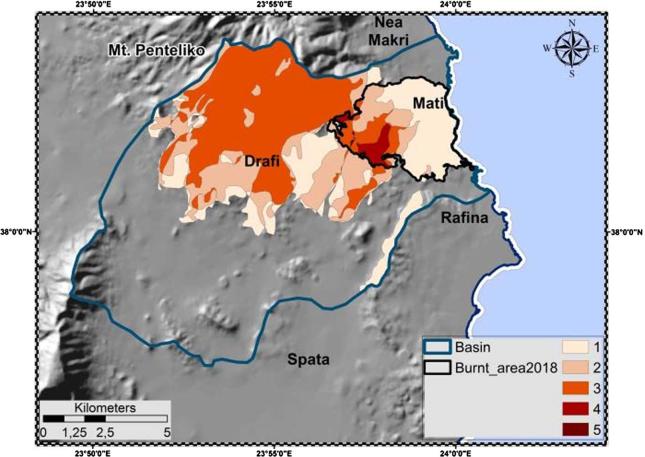
Historical fires overlay; fire frequency.

The four archive L5 TM images were co-registered and used for the detailed mapping of the historical wildfires that outburst in the study area. The most vulnerable areas are met where such fires have previously occurred, at cites displaying the highest fire frequency rate (Fig. 12). Apparently, changes in vegetation structure and community following repeated fires, can ultimately impact erosional processes and ecosystem recovery (Wittenberg and Inbar, 2009).

At these sites, the attributes of the model's individual factors, i.e. high-class R-factor (greater erosive potential of rainfall); LS-factor (rough morphology); C-factor (low vegetation cover); K-factor (greater soil erodibility) values and their complex combinations/inter-relations result in significant gross erosion rates. More specifically, the spatial patterns and numeric values of R-factor and erosive rainfall coincide. At high vulnerability regions, precipitation depth exceeds 600 mm (Fig. 5). Moreover, the highest K-factor values come from samples classified in the “Moderate Fine” class (Clay Loam; Sandy Clay Loam texture), displaying moderate to high erodibility, manifesting in easy to detach particles and crust formation proneness on the soil surface that leads to reduced infiltration and increased overland flow potential. The latter represent low organic matter (Evans, 1980) and/or high silt (Richter and Negendank, 1977) [or high clay (Evans, 1980)] content soils, characterized by low stone coverage and moderate infiltration (Table 6). The highest “moisture content at field capacity or 1/3 bar tension” (MS) and “bulk density of the top soil layer” (BD) values [high BD decreases infiltration and water holding capacity, leading to increased runoff potential; a BD increase can obstruct water (air; nutrients) movement (Doran, 2002)] are attained by the “Moderate Fine” and “Fine” soils (Table 6), leading to low soil moisture storage capacity and by extension high runoff. Furthermore, the highest cohesion (COH) is met on the “Moderate Fine” and “Fine” soils (the lowest on the “Moderate Coarse” ones). These textural classes display resistance to erosion i.e. less particle detachment by runoff [expressed as inverse function of COH (Quansah, 1982, Rauws and Govers, 1988)]. Contrary, the lowest K-factor values derive from the “Moderate Coarse” classes samples, at high organic matter content; stone cover percentage cites [large coarse fragments are less susceptible to transfer by being heavier; fine particles are detached more difficultly, by having higher cohesion strength (Morgan, 2005)]. Additionally, the highest LS-factor values (>15) were found at the high relief areas (hillslopes) of the basin (Fig. 2). It is here that the increased slope length (L) and steepness (S) account for greater runoff volume and velocity respectively, leading to a more intensive detachment of soil particles and thus a higher erosion rate (Haan et al., 1994). Finally, the highest C-factor characterizes low or moderate land cover areas, i.e. mainly on “*sparsely vegetated areas*” and less (comparatively) on “*vineyards*” (CLC coding). The basin's forested mountainous areas are assigned with the lowest C-factor values. The canopy cover density; height (above-ground features) are related to rainfall interception while the ground cover; rooting system (on- and below-ground features) to runoff generation.

**Table 6:** MS, BD, COH parameters' indicative values per textural class (Morgan, 2001, Morgan and Duzant, 2008).

Texture	Class	MS^1^ (%w w–^1^)	BD^2^ (Mg m–^3^)	COH^3^ (kPa)
Coarse	Sand (S)	0.08	1.5	2
	Loamy sand (LS)	0.15	1.4	2
Moderate coarse	Sandy loam (SL)	0.28	1.2	2
Medium	Loam (L)	0.20	1.3	3
	Silt (Si)	0.15	1.3	–
	Silty loam (SiL)	0.35	1.3	3
Moderate fine	Sandy clay loam (SCL)	0.38	1.4	3
	Clay loam (CL)	0.40	1.3	10
	Silty clay loam (SiCL)	0.42	1.3	9
Fine	Sandy clay (SC)	0.28	1.4	–
	Silty clay (SiC)	0.30	1.3	10
	Clay (C)	0.45	1.1	12

1 Moisture content at field capacity or 1/3 bar tension.

2 Bulk density of the top soil layer.

3 Cohesion.

Results are also affected by other parameters, like RUSLE's shortcomings, the rainfall characteristics i.e. inter-annual height; intensity and the mean annual fluctuation of precipitation, and the description of the soil characteristics based on soil samples. RUSLE's limitations are described in detail in Section 2.3.1. Rainfall fluctuation provokes analogous variation on the R-factor, affecting its multi-annual value. Moreover, soil samples information is limited to a local; point interest, the samples are small in numbers and unevenly distributed and they are spatially reduced based on a geostatistical method, which is an insufficient delineation given the high heterogeneity of soil attributes. In addition, the Desmet and Govers (1995) algorithm is a contributing area (CA) based method, using the Multiple Flow Direction (MFD) approach to estimate the LS-factor at the respective EU dataset. According to the latter, the slope length *λ* to CA conversion is performed considering two-dimensional topography, thus is not able to predict the cut-off conditions (Zhang et al., 2017) i.e. when a new segment begins as the slope gradient decreases. Furthermore, C-factor estimation was made by ascribing literature values (uniform per class) to the CLC base map. The stationary delineation of the cover management factor makes this approach inadequate to describe vegetation variation at large scales (Wang et al., 2002). Additionally, this methodology (I) corresponds to a very coarse spatial resolution (1:100,000) and therefore is not suitable for local land management planning, and (II) is characterized by several approximations and inconsistencies, i.e. generalization and broad grouping, classification errors. Moreover, as erosion is a continuous and episodic process, the seasonal variability of vegetation affects erosion rates and estimation accuracy. Therefore, the use of the CLC, given its static nature, is unable to provide multi-temporal information, and only leads to approximate erosion estimates that entirely neglect the dynamic character of this episodic phenomenon in diverse landscapes. Alexandridis et al. (2015) used variable time points to estimate the C-factor, underlining the significant contrast in the estimation of soil loss when considering the seasonal alterations of vegetation.

### 3.4 Post-fire analysis

The post-fire application of RUSLE yielded mean annual soil loss of 5.98 t ha^−1^ y^−1^, having variability range of 3.44–149.54 t ha^−1^ y^−1^. The most erosion-prone areas (coloured red) are also situated at the north of the basin [Fig. 11(b)], yet contrary to the pre-fire state they have expanded to include a notable part of the Mati settlement. The identical pre-; post-fire minimum and maximum gross erosion values indicate that they are still met outside the WUI zone. In the post-fire analysis, the inclusion of the area affected by the expansion of the fire within the settlement boundaries resulted in a greater mean annual soil loss. Focusing on the burnt area, RUSLE yielded mean annual soil loss of 18.58 t ha^−1^ y^−1^, having variability range of 0.00–135.88 t ha^−1^ y^−1^. Mean annual and maximum soil loss values are notably increased at the post-fire delineation (five and two times higher, respectively), with the moderate to high-risk regions (red colour) occupying almost the entire settlement area [Fig. 11(d)]. Although the burnt area comprises only 10.7% (14.49 km^2^; Table 5) of the basin, the numeric results along with the spatial expansion of the erosion-prone cites constitute a strong index of the wildfire's devastating effects. As in the pre-fire state, RUSLE showed large variations in erosion rates at the study site.

The linear character of the model, and the fixed state of the R-; K-; LS-; P-factors per application, lead to the conclusion that the discrepancies in the pre; post-fire estimations are “controlled” only by the different C-factor values, i.e. the density and spatial distribution of vegetation cover. According to Wang et al. (2002) RUSLE displays high sensitivity to C-factor alterations, particularly when modelling post-fire erosion (Larsen and MacDonald, 2007). Several attempts have been made to assign post-fire C-factor values [e.g. 0.2 (Larsen and MacDonald, 2007, Rulli et al., 2013), 0.25 (Fernandez et al., 2010), 0.27 (Gonzalez-Bonorino et al., 2003), 0.35–0.55 (Lykoudi and Zarris, 2002), 0.9 (Di Piazza et al., 2007)]. At the present study, the base value of 0.55, introduced after a European literature review by Panagos et al. (2015c), was modified i.e. weighted to 0.48 (Table 5). This post-fire C-factor tends to decrease exponentially after the first year of the fire towards a value of pre-fire conditions at the fourth year after the event (Borrelli et al., 2016). This happens provided that the land recovers the same cover type (e.g. forest, shrubland, etc) as the one before the fire.

Furthermore, burn severity is classified among the most significant variables in triggering alterations on soil loss and runoff response (Fox et al., 2008) after a fire event. NBR was used as the main (proxy) index for assessing such alterations. As results showed (Fig. 8), extended portions of the WUI were almost completely affected by the fire leading to lower (low NBR indicates bare ground and recently burned areas) local and mean NBR values. The latter interpret the extremeness of the event that almost wiped out the entire settlement. Overall, the post-fire NBR and soil loss maps “coincide”, supporting the general theoretical principals, according to which in areas of high burn severity instability and soil erosion have greater probability to manifest (Benavides-Solorio and MacDonald, 2001).

Moreover, bedrock within the WUI zone [Fig. 2(b)] comprises almost entirely Neogene formations. They are characterized by moderately high to high susceptibility to erosion, favouring surface runoff. The post-fire loss of vegetation cover has exposed these formations to the erosive powers of precipitation (raindrop impact; surface runoff), leading towards accelerated rates of soil erosion. Yet, although increased runoff (in terms of both volume and velocity), unhindered transition downstream and manifestation of flooding events are possible, sedimentation rate is expected to be restrained to realistic levels by the settlement's spatial configuration, acting as a natural barrier. Limestones are also present, at the north-northwest part of the burnt area and display little susceptibility to erosion due to increased water infiltration. Their effect on erosion is almost negligible, due to their high permeability and limited extent.

Change in soil properties that affect erosion processes, such as sealing i.e. the clogging of soil pores that decreases soil water holding capacity and infiltration, and leads to accelerated storm water runoff and surface erosion, formation of water repellent layer, decrease in the effective hydrological depth (the soil depth within moisture storage capacity “regulates” the generation of overland flow), should also be taken into consideration when addressing the phenomenon's adverse effects, especially within the WUI zone.

### 3.5 Post-fire erosion control

The severity of fire effects on the basin's hydrologic response and erosion dynamics requires the development and application of an integrated prevention and mitigation strategy based on the accurate assessment of post-fire runoff and sediment yield. In general, erosion-control measures and rehabilitation plans mainly aim towards reclamation of soil cover, improved infiltration capacity restoration, reduction of sediment processes such as detachment and transport (Wohlgemuth et al., 2009, Fernandez et al., 2010, Myronidis et al., 2010), considering the severity of the problem and its socio-economic extensions (Boardman et al., 2003, Kontoes et al., 2009). The goal is to reduce the accelerated sediment yield until the regeneration of natural vegetation.

Erosion barriers, mulch, and chemical treatments should be applied to hillslopes. Erosion barriers contribute to the reduction of sediment volume reaching the waterbodies (or settlements met on the way), the decrease of runoff volume and velocity (by interrupting the straight pathway of overland flow downslope), and the increase of infiltration rates. Mulch treatment can “technically” increase ground cover on burned slopes prior to vegetation's natural regeneration, providing protection against splash-erosion and moreover enhancing soil stability (Robichaud et al., 2007). Restoration of soil drainage capacity (at least at burned croplands) could be achieved by ploughing or tilling, in order to break-up aggregates and/or the fire-induced water repellent layer (Keizer et al., 2008).

Drainage channel beds must be periodically cleaned of fire-induced wooden debris or sediments that have been transported to the basin's hydrographic network by (the currently increased) overland flow, to avoid blocking the drainage pathways and eventually overwhelming their capacity (Bocchiola et al., 2008).

Resources should also be focused on fuel reduction efforts, especially in the WUI (Husari et al., 2006). Biomass removal to reduce wildfire risk (or intensity, in case of outbreak) can produce highly effective short-term results (Agee and Skinner, 2005), regardless of the significant and recurring investments of time and money. Additional prevention actions should involve the use fire resistant building materials and clearing away the peripheral home ignition zone from flammable elements (Cohen, 2000).

In the aftermath of the wildfire event, the National Technical University of Athens presented a two-fold plan for the region, involving prevention and protection measures. The former, comprise the development of mixed forests over the burnt areas, with shrubs and trees, using passive refractory plants (e.g. laurel, vineyards, certain species of pine, beech, walnut, etc.) and the construction of residential control zones with appropriate vegetation at their perimeter. The latter comprise the creation of firefighting zones within the forest and the settlement (WUI zone), and several other measures and infrastructure improvements to tackle potential disasters in the future. The Geotechnical Chamber of Greece on the other hand focused on the preservation of the region's “green equilibrium” i.e. replacement of burnt forest; croplands; urban greenery by an equal amount of such elements, with possible changes in spatial planning.

## 4 Conclusions

The paper describes the utilization of innovative Earth Observation data and the application of the empirical RUSLE model at the Mati, Attika settlement Wildland-Urban Interface (WUI) zone and its antecedent basin, after the devastating fire of July 2018, for the simulation of soil erosion. The paper has successfully introduced the use of WUI land cover as an input for better estimating cover-management changes for soil erosion modelling.

Analysis comprised of two stages, one before the fire and one after it. Post-fire erosion rates are notably higher throughout the study area – and more specifically within the WUI zone. Furthermore, after the fire the high vulnerability cites occupy almost entirely the Mati settlement area. Remote sensing data were also used in order to determine fire severity. The derivative indices (Normalized Burnt Ratio Index etc.) post-fire values indicate the event's devastating effects on vegetation and soil. The sensitivity of cover-management factor is high in case of wildfire events propagating exponential increase of potential erosion rates in areas affecting by wildfires. The results of our study showed a 5-fold increase of erosion rates after the Mati wildfire compared to pre-fire conditions.

The realistic results attest that RUSLE can perform well at such “diverse” conditions (Mediterranean environment; post-fire; WUI zone), providing a solid basis for soil loss calculation and designation of high-risk erosion regions. Apart from its practical applicability, its simplicity, ease of use, low input data and computation demands are of equal importance. Moreover, the archive satellite data reveal how the repeated fires, can ultimately impact vegetation recovery or degradation and erosional processes.

While this research effort relied on the assessment of the soil degradational effect of the wildfire event in comparison to the previous unburnt state of the study area, alternative research pathways could also be sought. Such efforts could entail the improvement of post-fire soil loss rates accuracy by utilizing comprehensive; process-based erosion models (new field measurements and additional data need to be acquired for a more solid and objective analysis), the investigation of post-fire soil redistribution at different temporal scales through the application of tracers (e.g. ^137^Cs), the quantification of post-fire wind erosion (responsible for the redistribution of ash and fine minerogenic sediment), the investigation of fire impact on nutrient; organic matter content etc. losses (by extension soil fertility), carbon release and air pollution. Of high importance is the mapping of fuels by utilizing very high-resolution imagery (remote sensing applications), and their interactions with climatic, topographic, socio-economic etc. factors. The results can be included in a wide range of management practices, e.g. real-time support on fuel management during the fire season etc. Targeted efforts for the protection of the WUI could involve a review of the local agricultural policy in order to include the development (and spatial design) of *peri*-urban agricultural zones, towards controlling the growth of artificial areas and preventing the direct contact between woodland and urban land. Additional research on that matter could focus on exposure conditions (they vary depending on the fuels, terrain, weather, and characteristics of the community i.e. the housing density, the extent of community perimeter adjacent to wildlands) and structure vulnerability (components and materials used) towards reducing structure losses.

Overall, the study can serve as a preliminary guide for scientific and policy making purposes, towards developing and applying a post-fire soil management/erosion mitigation/restoration and protection plan in the area. Such plan(s) could involve technical (sediment retention dams etc.) and administrative measures (hydrologic design etc.), tackling the phenomenon's adverse and multifaceted effects. The latter could easily be integrated into a holistic assessment of disturbance agents responsible for land degradation, such as land use change, cultivation practices etc. It is underlined that research ought not to be confined on the scientific community but conveyed to the general public, raising awareness on fire-induced soil erosion.

## Declaration of Competing Interest

The authors declare that they have no known competing financial interests or personal relationships that could have appeared to influence the work reported in this paper.

## References

[b0005] Agee J.K., Skinner C.N. (2005). Basic principles of forest fuel reduction treatments. Forest Ecol. Manag..

[b0010] Alexakis D. (2008). Geochemistry of stream sediments as a tool for assessing contamination by arsenic, chromium and other toxic elements: East Attica region. Greece. European Water.

[b0015] Alexander P.M., Stewart S.I., Mockrin M.H., Keuler N.S. (2015). The relative impacts of vegetation, topography and spatial arrangement on building loss to wildfires in case studies of California and Colorado. Landscape Ecol..

[b0020] Alexandridis T.K., Sotiropoulou A.M., Bilas G., Karapetsas N., Silleos N.G. (2015). The effects of seasonality in estimating the C-factor of soil erosion studies. Land Degrad. Dev..

[b0025] Andreu V., Imeson A.C., Rubio J.L. (2001). Temporal changes in soil aggregates and water erosion after a wildfire in a Mediterranean pine forest. CATENA.

[b0030] Angulo-Martinez M., Lopez-Vicente M., Vicente-Serrano S.M., Beguerıa S. (2009). Mapping rainfall erosivity at a regional scale: a comparison of interpolation methods in the Ebro Basin (NE Spain). Hydrol. Earth Syst. Sci..

[b0035] Arnold J.G., Srinivasan R., Muttiah R.S., Williams J.R. (1998). Large area hydrologic modeling and assessment part I: model development. J. Am. Water Resour. Assoc..

[b0040] Athanasakis G., Psomiadis E., Chatziantoniou A. (2017). High-resolution Earth Observation data and spatial analysis for burn severity evaluation and post-fire effects assessment in the Island of Chios, Greece. Proceedings of SPIE 10428, Remote Sensing- Earth Resources and Environmental Remote Sensing/GIS Applications VII Poland, Warsaw.

[b0045] Beasley D.B., Huggins L.F., Monke E.J. (1980). ANSWERS: A model for watershed planning. T. ASAE.

[b0050] Benavides-Solorio J., MacDonald L.H. (2001). Post-fire runoff and erosion from simulated rainfall on small plots, Colorado front range. Hydrol. Process..

[b0055] Boardman J., Poesen J., Evans R. (2003). Socio-economic factors in soil erosion and conservation. Environ. Sci. Pol..

[b0060] Bocchiola D., Rulli M.C., Rosso R. (2008). A flume experiment on the formation of wood jams in rivers. Water Resour. Res..

[b0065] Borrelli P., Panagos P., Langhammer J., Apostol B., Schütt B. (2016). Assessment of the cover changes and the soil loss potential in European forestland: first approach to derive indicators to capture the ecological impacts on soil-related forest ecosystems. Ecol. Indic..

[b0070] Brakensiek D.L., Rawls W.J., Stephenson G.R. (1986). Determining the saturated hydraulic conductivity of a soil containing rock fragments. Soil Sci. Soc. Am. J..

[b0075] Brown L.C., Foster G.R. (1987). Storm erosivity using idealized intensity distributions. T. ASAE.

[b0080] Cerda A. (1998). Changes in overland flow and infiltration after a rangeland fire in Mediterranean scrubland. Hydrol. Process..

[b0085] Certini G. (2005). Effects of fire on properties of forest soils: a review. Oecologia.

[b0090] Clapp R.B., Hornberger G.M. (1978). Empirical equations for some soil hydraulic properties. Water Resour. Res..

[b0095] CLC, 2018. (accessed 24 January 2019). https://land.copernicus.eu/pan-european/corine-land-cover/clc2018.

[b0100] Cohen J.D. (2000). Preventing disaster—home ignitability in the wildland-urban interface. J. Forest..

[b0105] De la Rosa J.M., Gonzalez-Perez J.A., Gonzalez-Vazquez R., Knicker H., Lopez-Capel E., Manning D.A.C., Gonzalez-Vila F.J. (2008). Use of pyrolysis/GC-MS combined with thermal analysis to monitor C and N changes in soil organic matter from a Mediterranean fire affected forest. CATENA.

[b0110] Desmet P., Govers G. (1995). GIS-based simulation of erosion and deposition patterns in an agricultural landscape: a comparison of model results with soil map information. CATENA.

[b0115] Diaz-Pacheco J., Gutierrez J. (2014). Exploring the limitations of CORINE Land Cover for monitoring urban land-use dynamics in metropolitan areas. J. Land Use Sci..

[b0120] Di Piazza G.V., Di Stefano C., Ferro V. (2007). Modelling the effects of a bushfire on erosion in a Mediterranean basin. Hydrol. Sci. J..

[b0125] Doran J.W. (2002). Soil health and global sustainability: translating science into practice. Agr. Ecosyst. Environ..

[b0130] Efthimiou N., Lykoudi E., Karavitis C. (2014). Soil erosion assessment using the RUSLE model and GIS. European Water.

[b0135] Efthimiou N., Psomiadis E. (2018). The significance of land cover delineation on soil erosion assessment. Environ. Manage..

[b0140] Esteves T.C.J., Ferreira A.J.D., Ferreira C.S.S., Bento C.P.M., Carreiras M.A., Soares J.A.A., Coelho C.O.A., Kirkby M.J., Irvine B.J., Shakesby R.A. (2012). Mitigating land degradation caused by wildfire: application of the PESERA model to fire-affected sites in central Portugal. Geoderma.

[b0145] EU Science hub news, 2018. JRC supports wildfire monitoring in the EU. (Accessed: 15.7.2019). https://ec.europa.eu/jrc/en/news/jrc-supports-wildfire-monitoring-eu.

[b0150] European Commission (EC), 2002. Forest Fires in Europe: 2001 Fire Campaign. Report No 2, European Communities, Italy.

[b0155] European Environment Agency (EEA), 2007. European forest types: categories and types for sustainable forest management reporting and policy. Technical report No 9/2006, Office for official publications of the European Communities, Luxemburg.

[b0160] Evans R., Kirkby M.J., Morgan R.P.C. (1980). Mechanics of water erosion and their spatial and temporal controls: an empirical viewpoint. Soil erosion.

[b0165] Food and Agriculture Organization (FAO), 2002. Guidelines on Fire Management in Temperate and Boreal Forests. Working Paper FP/1/E. Forest Resources Division, Rome.

[b0170] Fernandez C., Vega J.A., Vieira D.C.S. (2010). Assessing soil erosion after fire and rehabilitation treatments in NW Spain: Performance of RUSLE and revised Morgan–Morgan–Finney models. Land Degrad. Dev..

[b0175] Fox D.M., Maselli F., Carrega P. (2008). Using SPOT images and field sampling to map burn severity and vegetation factors affecting post forest fire erosion risk. CATENA.

[b0180] Frantzova A.F. (2012). Detecting and monitoring of wildfires with remote sensing data. South-Eastern European J. Earth Observat. Geomatics.

[b0185] Gao B.C. (1996). NDWI – A normalized difference water index for remote sensing of vegetation liquid water from space. Remote Sens. Environ..

[b0190] Gavrilovic S. (1962). A method for estimating the average annual quantity of sediments according to the potency of erosion. Bullet. Faculty Forestry.

[b0195] Gimeno-Garcia E., Andreu V., Rubio J.L. (2000). Changes in organic matter, nitrogen, phosphorus and cations in soil as a result of fire and water erosion in a Mediterranean landscape. Eur. J. Soil Sci..

[b0200] Gonzalez-Bonorino G., Osterkamp W.R., Pinol C. (2003). An averaging procedure for applying the Revised Universal Soil Loss Equation (RUSLE) to disturbed mountain watersheds. Geogaceta.

[b0205] Grammatikogiannis A., Mamassis N., Baltas E., Mimikou M. (2005). A meteorological telemetric network for monitoring of the Athens wider area (METEONET). A real time approach from point to areal measurements. Proceedings of the Ninth International Conference on Environmental Science and Technology (9CEST) Rhodes Island, Greece 2005.

[b0210] Gyssels G., Poesen J., Bochet E., Li Y. (2005). Impact of plant roots on the resistance of soils to erosion by water: a review. Prog. Phys. Geogr..

[b0215] Haan C.T., Barfield B.J., Hayes J.C. (1994). Design hydrology and sedimentology for small catchments.

[b0220] Hammer R.B., Stewart S.I., Radeloff V.C. (2009). Demographic trends, the Wildland-Urban Interface, and wildfire management. Soc. Natur. Resour..

[b0225] Husari S., Nichols H.T., Sugihara N.G., Stephens S.L., Sugihara N.G., van Wagtendonk J.W., Shaffer K.E., Fites-Kaufman J., Thode A.E. (2006). Fire and Fuel Management. Fires in California’s Ecosystems.

[b0230] Karali A., Hatzaki M., Giannakopoulos C., Roussos A., Xanthopoulos G., Tenentes V. (2013). Sensitivity and evaluation of current fire risk and future projections due to climate change: the case study of Greece. Nat. Hazards Earth Syst. Sci..

[b0235] Karamesouti M., Petropoulos G.P., Papanikolaou I.D., Kairis O., Kosmas K. (2016). Erosion rate predictions from PESERA and RUSLE at a Mediterranean site before and after a wildfire: Comparison & implications. Geoderma.

[b0240] Keeley J.E. (2009). Fire intensity, fire severity and burn severity: A brief review and suggested usage. Int. J. Wildland Fire.

[b0245] Keizer J.J., Doerr S.H., Malvar M.C., Prats S.A., Ferreira R.S.V., Õnate M.G., Coelho C.O.A., Ferreira A.J.D. (2008). Temporal variation in topsoil water repellency in two recently burnt eucalypt stands in north-central Portugal. CATENA.

[b0250] Kirkby M.J., Kirkby M.J., Morgan R.P.C. (1980). Modelling water erosion processes. Soil Erosion.

[b0710] Kirkby, M., Gobin, A., Irvine, B., 2003. Pan European Soil Erosion Risk Assessment. Deliverable 5: PESERA Model Strategy, Land Use and Vegetation Growth. European Soil Bureau (available at: http://eusoils.jrc.it/).

[b0255] Kontoes C.C., Poilve H., Florsch G., Keramitsoglou I., Paralikidis S. (2009). A comparative analysis of a fixed thresholding vs. a classification tree approach for operational burn scar detection and mapping. Int. J. Appl. Earth Obs. Geoinf..

[b0260] Kozanis S., Markonis Y. (2009). Hydrognomon Version 4 - User manual.

[b0265] Larsen I.J., MacDonald L.H. (2007). Predicting postfire sediment yields at the hillslope scale: testing RUSLE and disturbed WEPP. Water Resour. Res..

[b0270] Lillesand T.M., Kiefer R.W., Chipman J.W. (2004). Remote Sensing and Image Interpretation.

[b0275] López-Garcia M.J., Caselles V. (1991). Mapping burns and natural reforestation using thematic mapper data. Geocarto Int..

[b0280] Lu D., Li G., Valladares G.S., Batistella M. (2004). Mapping soil erosion risk in Rondônia, Brazilian Amazonia: using RUSLE, remote sensing and GIS. Land Degrad. Dev..

[b0285] Lykoudi E., Zarris D. (2002). Identification of regions with high risk of soil erosion in the island of Cephalonia using the Universal Soil Loss Equation. Proceedings of the Sixth National Conference of the Geographical Society of Greece, Volume II, 36 October, Thessaloniki, Greece.

[b0290] Mallik A.U., Gimingham C.H., Rahman A.A. (1984). Ecological effects of heather burning: I. Water infiltration, moisture retention, and porosity of surface soil. J. Ecol..

[b0295] Mallinis G., Mitsopoulos I., Dimitrakopoulos A., Gitas I., Karteris M. (2008). Integration of local scale fuel type mapping and fire behaviour prediction using high spatial resolution imagery. IEEE J. Sel. Topics Appl. Earth Observ..

[b0300] Mallinis G., Maris F., Kalinderis I., Koutsias N. (2009). Assessment of post-fire soil erosion risk in fire-affected watersheds using remote sensing and GIS. GISsci. Remote Sens..

[b0305] Mallinis G., Gitas I.Z., Tasionas G., Maris F. (2016). Multitemporal monitoring of land degradation risk due to soil loss in a fire-prone mediterranean landscape using multi-decadal landsat imagery. Water Resour. Manage..

[b0310] Marques M.A., Mora E. (1992). The influence of aspect on runoff and soil loss in a Mediterranean burnt forest (Spain). CATENA.

[b0315] Martınez J., Vega-Garcia C., Chuvieco E. (2009). Human-caused wildfire risk rating for prevention planning in Spain. J. Environ. Manage..

[b0320] Martinis S., Caspard M., Plank S., Clandillon S., Haouet S. (2017). Mapping burn scars, fire severity and soil erosion susceptibility in Southern France using multisensoral satellite data. IGARSS.

[b0325] Massada A., Redeloff V., Stewart S., Hawbaker T. (2009). Wildfire risk in the wildland–urban interface: a simulation study in northwestern Wisconsin. For. Ecol. Manage..

[b0330] McCool D.K., Brown L.C., Foster G.R. (1987). Revised slope steepness factor for the universal soil loss equation. T. ASAE.

[b0335] McCool D.K., Foster G.R., Mutchler C.K., Meyer L.D. (1989). Revised slope length factor for the universal soil loss equation. T. ASAE.

[b0340] Mell W.E., Manzello S.L., Maranghides A., Butry D., Rehm R.G. (2010). The wildland–urban interface fire problem – current approaches and research needs. Int. J. Wildland Fire.

[b0345] Mettos A., Ioakim Ch., Rondoyanni Th. (2000). Palaeoclimatic and palaeogeographic evolution of Attica-Beotia (Central-Greece). Special Publicat.-Geolog. Soc. Greece.

[b0350] Mitsopoulos I., Mallinis G., Arianoutsou M. (2015). Wildfire risk assessment in a typical Mediterranean wildland-urban interface of Greece. Environ. Manage..

[b0355] Modugno S., Balzter H., Cole B., Borrelli P. (2016). Mapping regional patterns of large forest fires in Wildland-Urban Interface areas in Europe. J. Environ. Manage..

[b0360] Moody J.A., Shakesby R.A., Robichaud P.R., Cannon S.H., Martin D.A. (2013). Current research issues related to post-wildfire runoff and erosion processes. Earth-Sci. Rev..

[b0365] Moreira F., Rego F.C., Ferreira P.G. (2001). Temporal (1958–1995) pattern of change in cultural landscape of northwestern Portugal: implication for fire occurrence. Landsc. Ecol..

[b0370] Morgan R.P.C., Quinton J.N., Smith R.E., Govers G., Poesen J.W.A., Auerswald K., Chisci G., Torri D., Styczen M.E. (1998). The European Soil Erosion Model (EUROSEM): A dynamic approach for predicting sediment transport from fields and small catchments. Earth Surf. Proc. Land..

[b0375] Morgan R.P.C. (2001). A simple approach to soil loss prediction: A revised Morgan-Morgan-Finney model. CATENA.

[b0380] Morgan R.P.C. (2005). Soil erosion and conservation.

[b0385] Morgan R.P.C., Duzant J.H. (2008). Modified MMF (Morgan-Morgan-Finney) model for evaluating effects of crops and vegetation cover on soil erosion. Earth Surf. Proc. Land..

[b0390] Mouillot F., Rambal S., Joffre R. (2002). Simulating climate change impacts on fire frequency and vegetation dynamics in a Mediterranean-type ecosystem. Glob. Change Biol..

[b0395] Myronidis D., Arabatzis G. (2009). Evaluation of Greek Post-Fire Erosion Mitigation Policy through Spatial Analysis. Pol. J. Environ. Stud..

[b0400] Myronidis D.I., Emmanouloudis D.A., Mitsopoulos I.A., Riggos E.E. (2010). Soil erosion potential after fire and rehabilitation treatments in Greece. Environ. Model. Assess..

[b0405] Nearing M.A., Foster G.R., Lane L.J., Finkner S.C. (1989). A process-based soil erosion model for USDA: water erosion prediction project technology. T. ASAE.

[b0410] Neary D.G., Klopatek C.C., DeBano L.F., Ffolliott P.F. (1999). Fire effects on belowground sustainability: A review and synthesis. For. Ecol. Manage..

[b0415] Neary, D.G., Ryan, K.C., DeBano, L.F. (Eds.), 2005 (revised 2008). Wildland fire in ecosystems: effects of fire on soils and water. Gen. Tech. Rep. RMRS-GTR-42-.4. Ogden, UT: US Department of Agriculture, Forest Service, Rocky Mountain Research Station, pp. 250.

[b0420] Neitsch S.L., Arnold J.G., Kinir J.R., Williams J.R. (2005). Soil and Water Assessment Tool. Theoretical Documentation, Version 2005.

[b0425] Orgiazzi A., Ballabio C., Panagos P., Jones A., Fernández-Ugalde O. (2018). LUCAS Soil, the largest expandable soil dataset for Europe: a review. Eur. J. Soil Sci..

[b0430] Panagos P., Meusburger K., Alewell C., Montanarella L. (2012). Soil erodibility estimation using LUCAS point survey data of Europe. Environ. Model. Softw..

[b0435] Panagos P., Meusburger K., Ballabio C., Borrelli P., Alewell C. (2014). Soil erodibility in Europe: A high-resolution dataset based on LUCAS. Sci. Total Environ..

[b0440] Panagos P., Borrelli P., Meusburger K. (2015). A new European slope length and steepness factor (LS-factor) for modeling soil erosion by water. Geosci. J..

[b0445] Panagos P., Borrelli P., Meusburger K., van der Zanden E.H., Poesen J., Alewell C. (2015). Modelling the effect of support practices (P-factor) on the reduction of soil erosion by water at European scale. Environ. Sci. Policy.

[b0450] Panagos P., Borrelli P., Meusburger C., Alewell C., Lugato E., Montanarella L. (2015). Estimating the soil erosion cover-management factor at European scale. Land Use Policy.

[b0455] Panagos P., Borrelli P., Poesen J., Ballabio C., Lugato E., Meusburger K., Montanarella L., Alewell C. (2015). The new assessment of soil loss by water erosion in Europe. Environ. Sci. Pol..

[b0460] Pausas J.G., Carbo E., Neus Caturla R., Gil J.M., Vallejo R. (1999). Postfire regeneration patterns in the eastern Iberian Peninsula. Acta Oecol..

[b0465] Pausas J.G., Paula S. (2012). Fuel shapes the fire-climate relationship: evidence from Mediterranean ecosystems. Global Ecol Biogeogr..

[b0470] Penman T.D., Bradstock R.A., Price O.F. (2014). Reducing wildfire risk to urban developments: simulation of cost effective fuel treatment solutions in south eastern Australia. Environ. Model. Softw..

[b0475] Petropoulos G.P., Kontoes C., Keramitsoglou I. (2011). Burnt area delineation from a uni-temporal perspective based on Landsat TM imagery classification using Support Vector Machines. Int. J. Appl. Earth Obs. Geoinf..

[b0480] Poesen J.W., Torri D., Bunte K. (1994). Effects of rock fragments on soil erosion by water at different spatial scales: a review. CATENA.

[b0485] Psomiadis E., Soulis K., Zoka M., Dercas N. (2019). Synergistic approach of remote sensing and GIS techniques for flash-flood monitoring and damage assessment in Thessaly plain area. Greece. Water.

[b0490] Quansah C. (1982). Laboratory experimentation for the statistical derivation of equations for soil erosion modelling and soil conservation design (Doctoral Dissertation).

[b0495] Rauws G., Govers G. (1988). Hydraulic and soil mechanical aspects of rill generation on agricultural soils. J. Soil Sci..

[b0500] Rawls W.J., Brakensiek C.L., Saxton K.E. (1982). Estimation of soil water properties. T. ASAE.

[b0505] Renard K.G., Foster G.R., Weesies G.A., Porter J.P. (1991). RUSLE: Revised Universal Soil Loss Equation. J. Soil Water Conserv..

[b0510] Renard K.G., Foster G.R., Weesies G.A., McCool D.K., Yoder D.C. (1996). Predicting Soil Erosion by Water: A Guide to Conservation Planning with the Revised Universal Soil Loss Equation.

[b0515] Richter G., Negendank J.F.W. (1977). Soil erosion processes and their measurement in the German area of the Moselle river. Earth Surf. Process..

[b0520] Robichaud P.R. (2000). Fire effects on infiltration rates after prescribed fire in Northern Rocky Mountain forests. USA. J. Hydrol..

[b0525] Robichaud P.R., Pierson F.B., Brown R.E., Wagenbrenner J.W. (2007). Measuring effectiveness of three postfire hillslope erosion barrier treatments, western Montana. USA. Hydrol. Process..

[b0530] Robichaud R.P., Cerda A., Cerda A., Robichaud P.R. (2009). Summary and remarks. Chapter 21. Fire Effects on Soils and Restoration Strategies.

[b0535] Rouse J.W., Haas R.H., Schell J.A., Deering D.W. (1973). Monitoring vegetation systems in the Great Plains with ERTS. Third ERTS symposium, NASA SP-351, 10–14 December, Washington DC, USA.

[b0540] Rulli M.C., Spada M., Bozzi S., Bocchiola D., Rosso R. (2006). Rainfall simulations on a fire disturbed Mediterranean area. J. Hydrol..

[b0545] Rulli M.C., Offeddu L., Santini M. (2013). Modeling post-fire water erosion mitigation strategies. Hydrol. Earth Syst. Sci..

[b0550] Sader S.A., Winne J.C. (1992). RGB-NDVI color composites for visualizing forest change dynamics. Int. J. Remote Sens..

[b0555] San-Miguel-Ayanz J., Schulte E., Schmuck G., Camia A. (2013). The European Forest Fire Information System in the context of environmental policies of the European Union. Forest Policy Economics.

[b0560] Shakesby R.A. (2011). Post-wildfire soil erosion in the Mediterranean: Review and future research directions. Earth Sci. Rev..

[b0565] Shanmugapriya P., Rathika S., Ramesh T., Janaki P. (2019). Applications of remote sensing in agriculture – A review. Int. J. Current Microbiol. Appl. Sci..

[b0570] Sifakis N.I., Iossifidis C., Kontoes C.C., Keramitsoglou I. (2011). Wildfire detection and tracking over greece using MSG, SEVIRI satellite data. Remote Sens.-Basel.

[b0575] Smets T., Poesen J., Bochet E. (2008). Impact of plot length on the effectiveness of different soil-surface covers in reducing runoff and soil loss by water. Prog. Phys. Geogr..

[b0580] Soil Science Division Staff (SSDS), 2017. Soil survey manual, in: Ditzler, C., Scheffe, K., Monger, H.C. (Eds.), USDA Handbook 18. US Government Printing Office, Washington DC.

[b0585] Soulis K.X., Dercas N., Valiantzas J.D. (2012). Wildfires impact on hydrological response – the case of Lykorrema experimental watershed. Global Nets J..

[b0590] Tanase M.A., Kennedy R., Aponte C. (2015). Radar Burn Ratio for fire severity estimation at canopy level: An example for temperate forests. Remote Sens. Environ..

[b0595] Theobald D., Romme W. (2007). Expansion of the US wildland–urban interface. Landsc. Urban Plan..

[b0600] US Department of Agriculture (USDA), 2014. Rainfall Intensity Summarization Tool (RIST). (accessed 23 October 2018). http://www.ars.usda.gov/News/docs.htm?docid=3251.

[b0610] Varela M.E., Benito E., Keizer J.J. (2010). Wildfire effects on soil erodibility of woodlands in NW Spain. Land Degrad. Dev..

[b0615] Vega J.A., Fernandez C., Fonturbel T. (2005). Throughfall runoff and soil erosion after prescribed burning in gorse shrubland in Galicia (NW Spain). Land Degrad. Dev..

[b0620] Vieira D.C.S., Serpa D., Nunes J.P.C., Prats S.A., Neves R., Keizer J.J. (2018). Predicting the effectiveness of different mulching techniques in reducing post-fire runoff and erosion at plot scale with the RUSLE, MMF and PESERA models. Environ. Res..

[b0625] Wang G., Wente S., Gertner G.Z., Anderson A. (2002). Improvement in mapping vegetation cover factor for the universal soil loss equation by geostatistical methods with Landsat Thematic Mapper images. Int. J. Remote Sens..

[b0630] Williams R.G., Sheridan J.M. (1991). Effect of measurement time and depth resolution on EI calculation. T. ASAE.

[b0635] Wischmeier W.H. (1959). A rainfall erosion index for a universal soil-loss equation. Soil Sci. Soc. Am. Pro..

[b0640] Wischmeier W.H., Johnson C.B., Cross B.W. (1971). A soil erodibility nomograph for farmland and construction sites. J. Soil Water Conserv..

[b0645] Wischmeier W.H. (1975). Estimating the Soil Loss Equation's Cover and Management Factor for Undisturbed Areas. Present and Prospective Technology for Predicting Sediment Yields and Sources.

[b0650] Wischmeier W.H., Smith D.D. (1978). Predicting Rainfall Erosion Losses. A Guide to Conservation Planning. USDA Agric. HB No. 537. Washington DC.

[b0655] Wohlgemuth P.M., Beyers J.L., Hubbert K.R., Cerda A., Robichaud P.R. (2009). Rehabilitation strategies after fire: The California, USA experience. Fire Effects on Soils and Restoration Strategies.

[b0660] Woolhiser D.A., Smith R.E., Goodrich D.C. (1990). KINEROS: A kinematic runoff and erosion model. Documentation and user manual. USDA-ARS-77. Washington DC.

[b0665] World Meteorological Organization, 1996. Guide to Meteorological instruments and methods of observation, sixth ed. WMO, Geneva.

[b0670] Wittenberg L., Inbar M. (2009). The role of fire disturbance on runoff and erosion processes - a long-term approach, Mt. Carmel case study. Israel. Geogr. Res..

[b0675] Xanthopoulos G. (1996). Greece: the 1995 forest fire season. Int. Forest Fire News (ECE/FAO).

[b0680] Xanthopoulos G. (2002). The forest fires of 1995 and 1998 on Penteli Mountain. , Proceedings of the International Workshop on “Improving Dispatching for Forest Fire Control”, 6–8 December, Chania, Greece.

[b0685] Xanthopoulos G. (2006). Fires in the urban-wildland interface area: a complex problem.

[b0690] Yassoglou N., Fantechi R., Peter D., Balabanis P., Rubio J.L. (1995). Land and desertification. Desertification in a European context: physical and socio-economic aspects.

[b0695] Yin S., Xie Y., Nearing M.A., Wang C. (2007). Estimation of rainfall erosivity using 5- to 60- minute fixed-interval rainfall data from China. CATENA.

[b0700] Young R.A., Onstad C.A., Bosch D.D., Anderson W.P. (1987). AGNPS, Agricultural non-Point-Source Pollution Model. A watershed analysis tool. USDA Conservation Research Report No.35. Washington DC.

[b0705] Zhang H., Wei J., Yang O., Baartman J.E.M., Gai L., Yang X., Li S., Yu J., Ritsema C.J., Geissen V. (2017). An improved method for calculating slope length (λ) and the LS parameters of the Revised Universal Soil Loss Equation for large watersheds. Geoderma.

